# Draft genome sequence of endophytic fungus *Talaromyces purpureogenus* strain YAFEF302, isolated from *Juglans sigillata*

**DOI:** 10.1128/mra.00829-23

**Published:** 2023-12-05

**Authors:** Yi Wang, Yuexian Yang, Xiaolong Yuan, Shanjun Ma, Dong Wang, Lu Qiao

**Affiliations:** 1 College of Landscape and Horticulture, Yunnan Forestry Technological College, Kunming Yunnan, China; 2 Laboratory of Forest Plant Cultivation and Utilization, Yunnan Academy of Forestry and Grassland, Kunming Yunnan, China; 3 The Key Laboratory of Biodiversity Conservation of Southwest China State Forestry Administration, College of Forestry, Southwest Forestry University, Kunming, China; University of California, Riverside, Riverside, California, USA

**Keywords:** *Talaromyces purpureogenus*, genome, *Juglans sigillata*

## Abstract

Here, we report a draft genome sequence of endophytic fungus *Talaromyces purpureogenus* strain YAFEF302, isolated from *Juglans sigillata*. The genome resource will support future research into potential secondary metabolite diversity of this fungus.

## ANNOUNCEMENT

Endophytic fungi are a kind of fungi living in the healthy plant tissues and organs (1). They have been considered as novel sources of bioactive metabolites with various biological activities, including anticancer (2), antimicrobial (3), antiviral (4), antifungal (5), and cytotoxic (6). Many new terpenoids, alkaloids, polyketides, phenolic compounds, coumarins, and quinone derivatives have been reported from endophytic fungi (7). *Talaromyces purpureogenus* is a special endophytic fungus isolated from both marine and terrestrial plants (8, 9). The extract of *T. purpureogenus* shows antiproliferative, antioxidative (10), antibacterial (9), and insecticidal (11) activity. In this study, we report the genome of *T. purpureogenus* strain YAFEF302, which has antimicrobial bioactivity against multi-drug-resistant pathogenic bacteria.

The endophytic fungus *T. purpureogenus* strain YAFEF302 was isolated from the bark of *Juglans sigillata*. The isolation process was as follows. The bark of *J. sigillata* was collected from Kunming arboretum, Yunnan Academy of Forestry and Grassland, Yunnan Province, China (25°14*'*14*"* N, 102°75*'*15*"* E) in 2020. The bark was washed by soaking it in tap water for 24 hours and then rinsing it with sterile water for 5 minutes. A tissue grinder was used to grind the bark, after which sterile water was added to obtain a slurry. Finally, the bark slurry was further diluted and spread onto modified potato dextrose agar (PDA) (12) with 100 µg/mL ampicillin, kanamycin, and streptomycin, respectively. After 4 days of incubation (28°C), single colonies were transferred to fresh PDA medium. One single colony (strain YAFEF302) was identified as *T. purpureogenus* by sequencing the ITS region, and the ITS sequence was deposited in GenBank (accession number: OR481912). *T. purpureogenus* strain YAFEF302 was deposited in the China Center for Type Culture Collection (deposit number: CCTCC M 2022612).

The mycelia of *T. purpureogenus* strain YAFEF302 were harvested for DNA extraction after cultivation at 28°C for 7 days on a rotary shaker at 150 rpm. Genomic DNA was extracted using a DNeasy Mini Kit (Qiagen, Valencia, CA, USA) following the manufacturer’s protocol. Illumina sequencing libraries were prepared using TruSeq DNA Sample Prep Kit (Illumina) with insert sizes of 400 bp. The library was sequenced on the Illumina NovaSeq platform with a 2 × 150-bp paired-end configuration. Total 25,297,004 raw reads were obtained (Table 1). The high-quality cleaned sequences (3.80 Gb; coverage, 136×) were obtained by trimming adapter sequences with AdapterRemoval version 2 (13). The genome was assembled using SPAdes version 3.10.1 (14). The genome assembly size of YAFEF302 was 28,865,709 bp with 52 scaffolds. The longest scaffold size was 3,053,887 bp, with an N50 value of 1,491,803 bp and an N90 value of 499,408 bp. The GC content was 45.63%. The gene completeness of genome assembly was assessed by using BUSCO version 5.4.2 (15), and the completeness value was 98.9%.

**TABLE 1:** Genome sequencing statistics

Parameter	Value
Raw reads	
Read number	25,297,004
Total base	3,819,847,604
Coverage	136×
Genome assembly	
Total base	28,865,709
Total scaffolds	52
Max sequence length	3,053,887
N50	1,491,803 bp
N90	499,408 bp
GC content	45.63%
Genome assembly assessment	
Complete BUSCOs	98.90%
Complete and single-copy BUSCOs	98.40%
Complete and duplicated BUSCOs	0.50%

## Data Availability

This study project is available under NCBI BioProject accession number PRJNA1011018 and BioSample accession number SAMN37208211. The complete genome assembly of *T. purpureogenus* is available under GenBank accession number JAVIAQ000000000. The Illumina NovaSeq sequencing raw data were deposited in GenBank under the accession number SRX21578354.

## References

[1] Wang Z , Wang L , Pan Y , Zheng X , Liang X , Sheng L , Zhang D , Sun Q , Wang Q . 2023. Research advances on endophytic fungi and their bioactive metabolites. Bioprocess Biosyst Eng 46:165–170. doi:10.1007/s00449-022-02840-7 36565343

[2] Hridoy M , Gorapi MZH , Noor S , Chowdhury NS , Rahman MM , Muscari I , Masia F , Adorisio S , Delfino DV , Mazid MA . 2022. Putative anticancer compounds from plant-derived endophytic fungi: a review. Molecules 27:296. doi:10.3390/molecules27010296 35011527 PMC8746379

[3] Caruso DJ , Palombo EA , Moulton SE , Zaferanloo B . 2022. Exploring the promise of endophytic fungi: a review of novel antimicrobial compounds. Microorganisms 10:1990. doi:10.3390/microorganisms10101990 36296265 PMC9607381

[4] Lacerda Í , Polonio JC , Golias HC . 2022. Endophytic fungi as a source of antiviral compounds. Chem Biodivers 19:e202100971. doi:10.1002/cbdv.202100971 35426966

[5] Deshmukh S , Gupta M , Prakash V , Saxena S . 2018. Endophytic fungi: a source of potential antifungal compounds. JoF 4:77. doi:10.3390/jof4030077 29941838 PMC6162562

[6] Uzma F , Mohan CD , Hashem A , Konappa NM , Rangappa S , Kamath PV , Singh BP , Mudili V , Gupta VK , Siddaiah CN , Chowdappa S , Alqarawi AA , Abd Allah EF . 2018. Endophytic fungi—alternative sources of cytotoxic compounds: a review. Front Pharmacol 9:309. doi:10.3389/fphar.2018.00309 29755344 PMC5932204

[7] Tiwari P , Bae H . 2022. Endophytic fungi: key insights, emerging prospects, and challenges in natural product drug discovery. Microorganisms 10:360. doi:10.3390/microorganisms10020360 35208814 PMC8876476

[8] Kumari M , Taritla S , Sharma A , Jayabaskaran C . 2018. Antiproliferative and antioxidative bioactive compounds in extracts of marine-derived endophytic fungus Talaromyces purpureogenus. Front Microbiol 9:1777. doi:10.3389/fmicb.2018.01777 30123207 PMC6085570

[9] Sharma A , Sagar A , Rana J , Rani R . 2022. Green synthesis of silver nanoparticles and its antibacterial activity using fungus Talaromyces purpureogenus isolated from Taxus baccata LINN. Micro and Nano Syst Lett 10. doi:10.1186/s40486-022-00144-9

[10] Keekan KK , Hallur S , Modi PK , Shastry RP . 2020. Antioxidant activity and role of culture condition in the optimization of red pigment production by Talaromyces purpureogenus KKP through response surface methodology. Curr Microbiol 77:1780–1789. doi:10.1007/s00284-020-01995-4 32328751

[11] Yue Y , Jiang M , Hu H , Wu J , Sun H , Jin H , Hou T , Tao K . 2022. Isolation, identification and insecticidal activity of the secondary metabolites of Talaromyces purpureogenus BS5. J Fungi 8:288. doi:10.3390/jof8030288 PMC8949156 35330290

[12] Santra HK , Banerjee D . 2022. Broad-spectrum antimicrobial action of cell-free culture extracts and volatile organic compounds produced by endophytic fungi Curvularia eragrostidis. Front Microbiol 13:920561. doi:10.3389/fmicb.2022.920561 35814705 PMC9260591

[13] Schubert M , Lindgreen S , Orlando L . 2016. Adapterremoval v2: rapid adapter trimming, identification, and read merging. BMC Res Notes 9:88. doi:10.1186/s13104-016-1900-2 26868221 PMC4751634

[14] Prjibelski A , Antipov D , Meleshko D , Lapidus A , Korobeynikov A . 2020. Using SPAdes de novo assembler. Curr Protoc Bioinform 70:e102. doi:10.1002/cpbi.102 32559359

[15] Simão FA , Waterhouse RM , Ioannidis P , Kriventseva EV , Zdobnov EM . 2015. BUSCO: assessing genome assembly and annotation completeness with single-copy orthologs. Bioinform 31:3210–3212. doi:10.1093/bioinformatics/btv351 26059717

